# Leader bottom-line mentality and employees’ innovative work behavior: the role of workplace spirituality and value congruence

**DOI:** 10.3389/fpsyg.2026.1912519

**Published:** 2026-09-01

**Authors:** Ying Zhou, Ting Li, Xiaoyue Li

**Affiliations:** 1 Henan Institute of Economics and Trade, Zhengzhou, China; 2 UCSI Graduate Business School, Cheras, Malaysia; 3 Beijing Union University, Beijing, China

**Keywords:** individual–organizational value congruence, innovative work behavior, leader bottom-line mentality, meaning-related work experience, workplace spirituality

## Abstract

**Introduction:**

Leader bottom-line mentality (BLM) may focus employees’ attention on measurable results, but it may also narrow the conditions under which they are willing to develop and implement new ideas. This study examines the indirect association between leader BLM and innovative work behavior (IWB) through workplace spirituality (WS) and tests whether individual-organizational value congruence (IOVC) moderates the BLM-WS association.

**Methods:**

Survey data from 355 Chinese employees aged 18-43 were analyzed using partial least squares structural equation modeling (PLS-SEM).

**Results:**

Leader BLM was negatively associated with WS, whereas WS was positively associated with IWB. In the structural model, the direct association between BLM and IWB was not significant, whereas the indirect association through WS was negative and significant. The BLM x IOVC interaction was also not significant, providing no support for the proposed buffering role of IOVC.

**Discussion:**

The findings were more consistent with a negative indirect association through employees’ experience of meaning, connection, and value alignment than with a direct negative association between BLM and IWB. The proposed buffering role of value congruence was not supported in this sample. Because the data were cross-sectional and collected from a single source, the results should be interpreted as associations rather than causal evidence.

## 1 Introduction

Formal innovation strategies are important, but many innovations begin in ordinary work situations: employees notice a problem, envision a better approach, and try to put that idea into practice (Liu et al., 2016; Wang and Wu, 2025). Following Scott and Bruce (1994) and Janssen (2000), this study defines innovative work behavior (IWB) as employees’ discretionary efforts to generate ideas, gain support for them, and implement them in practice. Such efforts are inherently uncertain: ideas may take time to develop, fail to attract support, or be rejected before their value becomes apparent (Javed et al., 2019; Wang and Lim, 2025). Employees’ experience of work is therefore relevant. They may be more willing to invest in uncertain ideas when they view their work as worthwhile, can rely on colleagues, and perceive their efforts as consistent with organizational values (Amabile and Pratt, 2016; Alfarajat and Emeagwali, 2021). Leaders also matter because employees often interpret everyday leadership signals as cues about which efforts are likely to be recognized, supported, or overlooked (Greenbaum et al., 2012; Mawritz et al., 2017).

These demands become especially consequential when leaders repeatedly emphasize bottom-line results. Leader bottom-line mentality (BLM) refers to a tendency to prioritize bottom-line outcomes over other organizational concerns. In everyday work, employees may interpret this orientation as signaling that numbers, targets, and visible results matter most (Greenbaum et al., 2012, 2023). Although such emphasis can clarify performance expectations, it may also leave less perceived room for learning, experimentation, and collaboration. Activities that do not produce immediate measurable returns may consequently appear less valued, making employees less inclined to invest time and psychological energy in ideas whose benefits are uncertain or delayed (Babalola et al., 2020).

Much of the BLM literature has examined associations between a narrow bottom-line focus and employees’ conduct, voice, interpersonal experiences, and performance-related responses (Babalola et al., 2020; Greenbaum et al., 2023). Emerging research has also linked BLM to innovation-related outcomes, often through risk, work values, or performance concerns (Greenbaum et al., 2020; Luo et al., 2025). However, less attention has been given to whether BLM is associated with employees’ experience of meaning and social value at work (Ren et al., 2018; Wang and Lee, 2023). This gap matters because innovation involves more than risk tolerance. Employees also need reasons to care about an idea, colleagues who can help advance it, and a sense that their efforts are consistent with the values emphasized by the organization (Amabile and Pratt, 2016; Hunsaker and Ding, 2022). The present study, therefore, examines the BLM–IWB relationship not only as a direct behavioral association but also in relation to employees’ broader experience of work.

To capture this broader work experience, the present study focuses on workplace spirituality (WS). Following Milliman et al. (2003), WS is conceptualized as an overarching experience reflected in meaningful work, a sense of community, and alignment with organizational values. Although these dimensions are conceptually distinct, they are expected to covary because each reflects a shared experience of purpose, connection, and value consistency at work. A strong bottom-line emphasis may be associated with lower WS when measurable output overshadows these aspects of work. Employees who report weaker meaning, connection, and value alignment may also be less willing to invest in ideas that require additional effort and social support (Alfarajat and Emeagwali, 2021; Durrah, 2023).

The model further examines whether value fit conditions employees’ responses to leader BLM. Individual–organizational value congruence (IOVC) refers to employees’ perceived compatibility between their own values and those emphasized by the organization (Kristof, 1996; Cable and DeRue, 2002; Edwards and Cable, 2009). Person–organization fit theory suggests that this compatibility may influence how employees interpret bottom-line cues, including whether they view such cues as legitimate or inconsistent with their personal values (Kristof, 1996; Edwards and Cable, 2009). When value fit is high, performance demands may be easier to understand as part of the organization’s broader direction; when it is low, the same demands may appear more detached from what employees personally value. However, this proposed moderating role requires careful treatment because IOVC is conceptually close to the alignment-with-organizational-values dimension of WS. Their distinctiveness is therefore examined empirically rather than assumed in advance (Cable and DeRue, 2002; Milliman et al., 2003).

**Figure 1:**
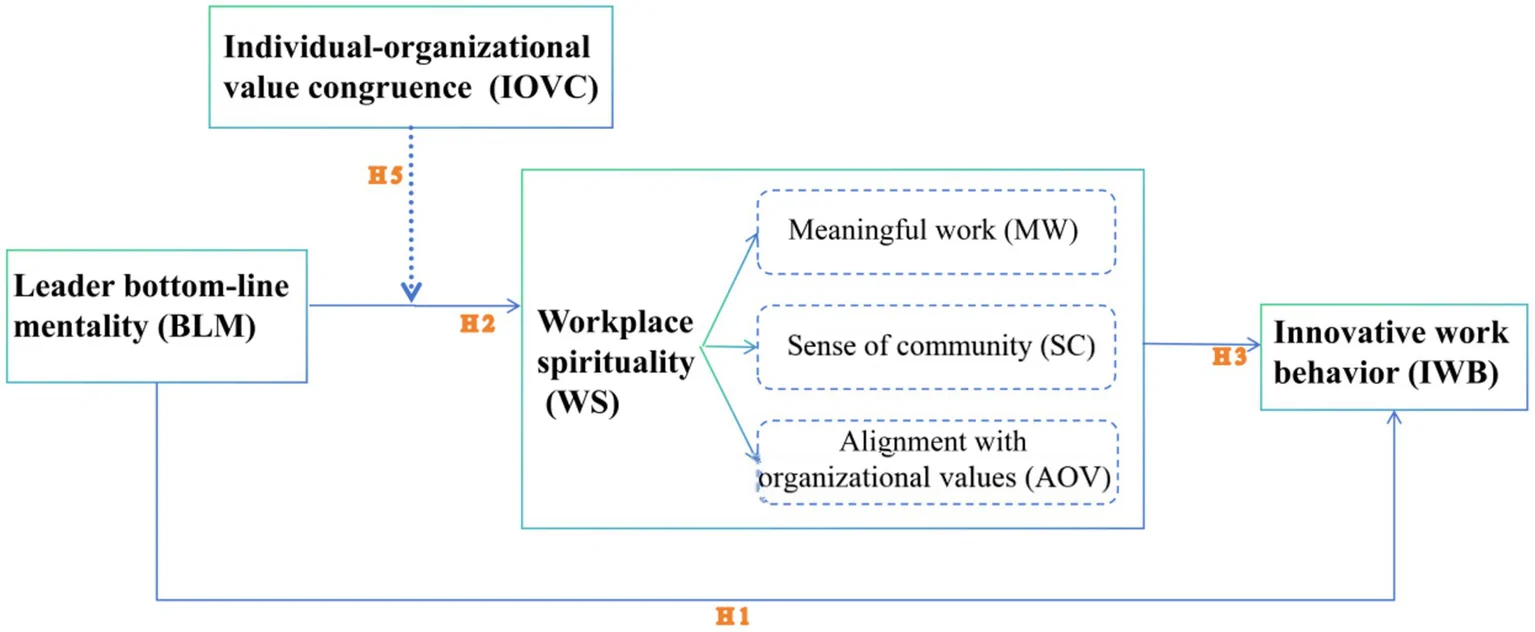
Research conceptual framework.

Against this background, the study addresses three questions: whether leader BLM is directly associated with employees’ IWB, whether it is indirectly associated with IWB through WS, and whether IOVC moderates the BLM–WS association. This framing examines employee innovation in BLM research not only in relation to risk and performance concerns but also in relation to employees’ experience of meaning, connection, and value consistency at work. It therefore positions WS as a work-experience construct that may be relevant to the observed BLM–IWB association (Eissa et al., 2020; Greenbaum et al., 2020; Riisla et al., 2021). The model also tests the proposed buffering role of IOVC while explicitly acknowledging the conceptual and measurement overlap between IOVC and the alignment-with-organizational-values dimension of WS (Figure 1).

## 2 Literature review and hypotheses

### 2.1 Theoretical background

#### 2.1.1 Leader bottom-line mentality as a social cue about work priorities

Following Greenbaum et al. (2012), leader BLM refers to a persistent tendency to prioritize bottom-line outcomes when other organizational concerns also require attention. It should not be understood as an occasional call for improved performance; rather, it reflects a recurring way in which leaders rank priorities, allocate attention, and evaluate success at work (Greenbaum et al., 2012, 2023). In practice, this orientation gives greater weight to measurable indicators such as financial results, productivity, and short-term performance metrics. For employees, leader BLM may therefore serve as a frame for judging which contributions are most likely to be recognized and valued by the organization.

Employees do not observe this orientation directly; rather, they infer it from recurring leadership cues embedded in everyday work. Evaluation practices, resource allocation, leader communication, reward decisions, and responses to mistakes can all signal which priorities are taken seriously (Khassawneh et al., 2022; Shi et al., 2024). When bottom-line cues are repeatedly emphasized, learning, collaboration, ethical reflection, interpersonal support, and long-term development may appear secondary. This pattern is particularly relevant to innovation because developing new ideas generally requires time, tolerance for uncertainty, and effort beyond routine task completion (Chen et al., 2022; Mawritz et al., 2024).

Viewing leader BLM as a social cue shifts attention beyond its association with immediate task performance (Salancik and Pfeffer, 1978; Greenbaum et al., 2023). A strong bottom-line emphasis may direct employees’ attention toward short-term measurable results while making meaning, connection, and shared values less salient in everyday work (Greenbaum et al., 2023; Liehr and Hauff, 2025). When success is repeatedly framed in narrow performance terms, employees may experience their work as less purposeful, less socially embedded, and less aligned with broader organizational values. This social-cue perspective therefore provides the basis for examining whether leader BLM is associated with workplace spirituality and, in turn, innovation-related behavior.

#### 2.1.2 Workplace spirituality as a meaning-related work experience

Workplace spirituality (WS) refers to employees’ experience of work as meaningful, socially connected, and consistent with organizational values (Milliman et al., 2003). The present study conceptualizes WS as a meaning-related work experience rather than as a proxy for basic psychological needs or intrinsic motivation. Its focus is on whether employees regard their work as purposeful, experience a sense of belonging in workplace relationships, and perceive compatibility between their own values and those emphasized by the organization.

Within this conceptualization, meaningful work, sense of community, and alignment with organizational values represent three related dimensions of WS. Meaningful work concerns the extent to which employees view their work as significant and worthwhile. Sense of community concerns feelings of connection, mutual respect, and belonging in workplace relationships. Alignment with organizational values concerns the perceived consistency between employees’ personal values and what the organization appears to stand for (Milliman et al., 2003; Afsar et al., 2016).

The reflective-reflective specification follows from this overarching experiential interpretation. Employees who report stronger WS are expected, on average, to report greater meaning in their work, stronger connection with others, and greater alignment with organizational values. The three dimensions are not assumed to be substantively identical; rather, they are modeled as correlated manifestations of a common underlying work experience (Sarstedt et al., 2019, 2022). The higher-order WS construct therefore captures the shared experiential variance across the three dimensions, while dimension-specific variance remains at the first-order level. Under this interpretation, no single dimension independently defines WS. Instead, variation in the broader WS experience is expected to be reflected across all three domains. This specification differs from a formative model, in which meaning, community, and value alignment would be treated as distinct components that jointly form the higher-order construct.

WS is relevant to the present model because the relationship between leader BLM and innovation may involve employees’ broader experience of work rather than only a direct behavioral association. When leaders repeatedly emphasize bottom-line outcomes, employees may perceive purpose, connection, and value consistency as less prominent in everyday work. Lower WS may, in turn, be associated with less willingness to invest discretionary effort in developing and advancing new ideas (Alfarajat and Emeagwali, 2021; Hunsaker and Ding, 2022).

#### 2.1.3 Innovative work behavior as discretionary and socially embedded behavior

Innovative work behavior (IWB) refers to a set of discretionary activities through which employees generate ideas, seek support for them, and help translate them into workplace practices (Scott and Bruce, 1994; Janssen, 2000). It is not merely an extension of assigned duties. Employees may need to identify a problem, envision an alternative way of working, persuade others of the idea’s value, and remain involved until the idea is implemented. IWB, therefore, involves uncertainty and social exposure because new ideas may fail, encounter resistance, or require time before their value becomes apparent (Janssen, 2000; Anderson et al., 2014).

Knowledge and skill alone may not fully explain why employees engage in IWB. Employees may also need a reason to invest additional effort, confidence that others will support the idea, and a sense that the idea is consistent with organizational values. Meaningful work can make improvement and problem solving feel worthwhile. A sense of community can facilitate idea sharing, help seeking, and collaboration when an idea encounters resistance. Alignment with organizational values can help employees view innovation as part of the organization’s broader direction rather than as an isolated personal initiative (Anderson et al., 2014; Hunsaker and Ding, 2022; Riaz, 2024).

These considerations clarify why WS may be relevant to IWB. Employees who report stronger WS may be more willing to invest discretionary effort in developing and advancing ideas. By contrast, when meaning, connection, and value alignment are weaker, innovation-related effort may appear less worthwhile, less supported, or less consistent with organizational priorities. The BLM–IWB relationship may therefore be better understood by considering employees’ experience of meaning, connection, and value alignment alongside the direct behavioral association (Alfarajat and Emeagwali, 2021; Hunsaker and Ding, 2022).

#### 2.1.4 Individual–organizational value congruence as a potential boundary condition

Drawing on person–organization fit research, individual–organizational value congruence (IOVC) refers to employees’ perceived compatibility between their personal values and the values emphasized by the organization (Cable and DeRue, 2002; Edwards and Cable, 2009; Kristof, 1996). This perceived compatibility may shape how employees interpret organizational practices and leadership cues. When value congruence is high, performance demands may be easier to understand as part of an acceptable organizational direction; when it is low, the same demands may appear less consistent with what employees personally value (Edwards and Cable, 2009).

This logic may be particularly relevant when employees encounter repeated bottom-line cues from their leaders. Employees with high IOVC may interpret these cues as less inconsistent with the organization’s broader direction because they already perceive substantial compatibility between their own values and those of the organization. Employees with low IOVC, by contrast, may perceive the same cues as more detached from their personal values and less supportive of a meaningful work experience. IOVC may therefore condition the strength of the association between leader BLM and workplace spirituality.

This proposed moderating role requires cautious treatment because IOVC is substantively close to the alignment-with-organizational-values (AOV) dimension of WS. In the present model, AOV is included as one dimension of the broader WS construct, whereas IOVC is specified as a separate perception of person–organization value compatibility (Cable and DeRue, 2002; Edwards and Cable, 2009; Milliman et al., 2003). This distinction concerns construct scope and structural role rather than entirely different substantive content. At the item level, both measures ask employees to evaluate the compatibility between their personal values and those associated with the organization. Assigning the constructs different positions in the model therefore does not create a sharp conceptual boundary between them. The present study retains both constructs because AOV forms part of the three-dimensional WS measure administered in the survey, but the moderation result is interpreted cautiously. First-order discriminant-validity assessment and sensitivity analyses excluding AOV are used as supplementary checks, and statistical separation is not treated as evidence of complete conceptual distinctiveness.

### 2.2 Hypotheses development

#### 2.2.1 Leader bottom-line mentality and innovative work behavior

From a social-cue perspective, leader BLM makes visible and measurable outcomes especially salient to employees. In everyday work, employees may come to view performance indicators, efficiency, and short-term results as the clearest evidence of valued contribution (Mawritz et al., 2017, 2024). Their attention and effort may consequently shift toward explicit targets and away from activities whose value is uncertain, delayed, or difficult to quantify (Babalola et al., 2020; Mawritz et al., 2024).

Innovative work behavior rarely produces immediate or easily measurable returns. It often involves experimentation, revision, and the possibility that an idea will fail or encounter resistance (Greenbaum et al., 2020). Compared with routine task performance, innovation may require employees to invest cognitive, emotional, and social effort before they know whether an idea will be accepted, supported, or rewarded. When bottom-line priorities are strongly emphasized, such effort may appear more difficult to justify, particularly when the expected benefits are uncertain or delayed (Luo et al., 2025).

Employees may therefore be less inclined to engage in exploratory or experimental activities that extend beyond immediate performance demands. Generating ideas, seeking support, and advancing new solutions may appear less worthwhile when short-term measurable outcomes dominate employees’ understanding of what the organization values.

*H1*: Leader BLM is negatively associated with employees’ IWB.

#### 2.2.2 Leader bottom-line mentality and workplace spirituality

In the present model, workplace spirituality reflects employees’ experience of work as meaningful, socially connected, and consistent with organizational values (Kim and Song, 2024; Sultan and Hussain, 2025). These perceptions may shift in response to recurring signals about what the organization appears to value. Employees often infer such priorities from what leaders communicate, reward, tolerate, and overlook in everyday work (Dik et al., 2024; Syahir et al., 2025).

Leader BLM may be associated with lower WS when bottom-line outcomes are emphasized more strongly than the meaning-related, relational, and value-based aspects of work. Repeated attention to financial results, productivity, and short-term performance indicators may make purpose, connection, learning, and value alignment appear secondary. Employees may consequently experience their work as less purposeful, less socially embedded, and less consistent with broader organizational values (Greenbaum et al., 2023; Baber et al., 2024).

A strong bottom-line emphasis may also create tension between measurable performance demands and employees’ understanding of the organization’s broader values. When only immediately quantifiable outcomes appear to count, employees may find it more difficult to connect their work with purposes and values beyond short-term results. Leader BLM is therefore expected to be negatively associated with WS.

*H2*: Leader BLM is negatively associated with WS.

#### 2.2.3 Workplace spirituality and innovative work behavior

Innovative work behavior requires employees to invest effort beyond their assigned tasks, often before the value of an idea becomes clear. Employees may need to identify opportunities for improvement, develop possible solutions, gain support from others, and help translate those ideas into practice (Scott and Bruce, 1994; Kim and Song, 2024). Such effort may be easier to sustain when employees regard their work as meaningful, feel supported in workplace relationships, and perceive their ideas as consistent with organizational values (Amabile and Pratt, 2016; Sode and Chenji, 2024).

WS is relevant to IWB because a stronger overall experience of workplace spirituality is expected to be reflected in meaningful work, a sense of community, and alignment with organizational values (Shalley et al., 2004; Dik et al., 2024). Meaningful work can give employees a reason to devote attention and energy to improvement and problem solving (Milliman et al., 2003; Dik et al., 2024). A sense of community can facilitate idea sharing, help seeking, and collaboration when an idea encounters resistance (Kim and Song, 2024). Alignment with organizational values can help employees view innovation as part of the organization’s broader direction rather than as an isolated or personally risky initiative (Kim and Song, 2024; Sode and Chenji, 2024).

Employees who report higher WS may, therefore, have a stronger experiential and relational basis for engaging in innovative behavior. When employees experience greater purpose, connection, and value alignment at work, they may be more willing to develop ideas, seek support for them, and help move them toward implementation.

*H3*: WS is positively associated with employees’ IWB.

#### 2.2.4 The mediating role of workplace spirituality

Bringing these arguments together, the model proposes a negative indirect association between leader BLM and employees’ IWB through WS (Scott and Bruce, 1994; Janssen, 2000; Kim and Song, 2024). When leader BLM signals that measurable outcomes receive priority over broader work concerns, employees may report weaker purpose, connection, and value alignment at work. As these experiences weaken, employees may become less willing to invest discretionary effort in developing and advancing new ideas (Shalley et al., 2004; Dik et al., 2024).

This mediation argument rests on two related associations. First, when bottom-line outcomes are emphasized over broader aspects of work, employees may experience their work as less meaningful, feel less connected to others, and perceive weaker alignment with organizational values (Milliman et al., 2003; Dik et al., 2024). Second, lower WS may be associated with less motivation and relational support to develop ideas, seek support for them, and help translate them into practice (Kim and Song, 2024).

WS is therefore expected to account for part of the association between leader BLM and IWB. This mediation argument does not replace the direct BLM–IWB association proposed in H1. Rather, it specifies an additional indirect association involving employees’ experience of meaning, connection, and value alignment.

*H4*: Leader BLM is indirectly associated with employees’ IWB through WS.

#### 2.2.5 The moderating role of individual–organizational value congruence

In the moderation argument, IOVC is treated as a value-fit condition: employees differ in the extent to which they perceive their personal values as compatible with those emphasized by the organization (Kristof, 1996; Paleń-Tondel, 2025). From a person–organization fit perspective, employees may interpret the same leadership cue differently. When IOVC is high, organizational demands may be easier to understand as part of a broader and acceptable organizational direction (Edwards and Cable, 2009; Lee et al., 2025).

This value-fit logic may be particularly relevant when leaders repeatedly communicate bottom-line priorities (Paleń-Tondel, 2025). Employees with high IOVC may interpret bottom-line demands as legitimate organizational priorities rather than as signals that their personal values are being displaced. Under these conditions, the negative association between leader BLM and employees’ experience of meaning, connection, and value alignment may be weaker. When IOVC is low, by contrast, the same bottom-line emphasis may appear more inconsistent with employees’ personal values and less supportive of a meaningful work experience (Lee et al., 2025).

The expected pattern is therefore a buffering one: higher IOVC is expected to weaken the negative association between leader BLM and WS, whereas lower IOVC is expected to leave this association stronger.

*H5*: IOVC moderates the negative association between leader BLM and WS, such that the association is weaker when IOVC is high.

## 3 Research methodology

### 3.1 Sample and procedure

This study employed a cross-sectional survey design. The target population comprised Chinese employees aged 18–43 who worked in organizations located in Henan Province, China. To ensure that respondents were familiar with their work setting and immediate supervisor, participation was restricted to employees who had worked in their current organization for at least 6 months and reported directly to a supervisor. Respondents holding managerial or executive positions were instructed to evaluate their own immediate supervisor rather than themselves.

The study was reviewed and approved by the Ethics Committee of Shaanxi Institute of International Trade & Commerce (Approval No. 2025051210). Before completing the questionnaire, participants were informed of the academic purpose of the study, the voluntary nature of participation, the anonymity of their responses, and their right to withdraw before submitting the questionnaire. Participants provided online informed consent on the first page of the survey.

Data were collected from 15 May to 31 July 2025, using SOJUMP, an online survey platform. To reduce duplicate participation, the platform was configured to accept only one submission from the same device. Participation was voluntary, and no monetary or non-monetary incentives were offered. A non-probability convenience sampling approach was adopted (Etikan et al., 2016). To reduce the possibility of dependence among responses, the recruitment protocol was designed to obtain no more than one eligible respondent from each participating organization. Each recruitment contact was instructed to invite only one eligible employee from the organization associated with that contact. Survey invitations were distributed through MBA student networks, human resource contacts, and professional contacts, primarily targeting employees in the financial services, technology, and manufacturing sectors in Henan Province. Eligible employees from other sectors were also included. Of the 520 potentially eligible employees invited to participate, 371 returned questionnaires, yielding an initial response rate of 71.35%.

The returned questionnaires were screened using four data-quality criteria. Completion times of less than 3 min were classified as unusually short. Straight-line responding was defined as selecting the same response option across all substantive scale items. Responses were also excluded when answers to the eligibility-screening questions were inconsistent with the corresponding demographic information, particularly age or organizational tenure, or when more than 10% of questionnaire responses were missing. Of the 16 excluded questionnaires, four were removed because of unusually short completion times, five because of straight-line responding, three because of inconsistencies between eligibility-screening responses and demographic information, and four because more than 10% of responses were missing. After these exclusions, 355 valid responses remained (Curran, 2016; Ward and Meade, 2023). The final valid response rate was 68.27% of invited employees, and the usable questionnaire rate among returned questionnaires was 95.69%.

Because the survey was anonymous, the analytical dataset did not retain organization- or supervisor-level identifiers. Consequently, although the recruitment protocol was intended to reduce the likelihood of recruiting multiple respondents from the same organization, it was not possible to independently verify whether each respondent represented a different organization or evaluated a different immediate supervisor. Potential clustering at the organization and supervisor levels could therefore not be assessed or modeled statistically. The analyses were accordingly conducted at the individual respondent level. Table 1 presents the demographic characteristics of the final sample.

**Table 1:** Demographic characteristics of respondents.

Characteristic	Category	*n*	%
Gender	Male	185	52.1
	Female	170	47.9
Age	18–23 years	84	23.7
	24–33 years	157	44.2
	34–43 years	114	32.1
Education	Secondary school certificate	68	19.2
	Diploma/technical school certificate	98	27.6
	Bachelor’s degree	152	42.8
	Master’s degree	37	10.4
Position	Non-managerial employee	213	60.0
	Manager	121	34.1
	Executive	21	5.9
Organizational tenure	6 months to 1 year	97	27.3
	1–5 years	94	26.5
	6–10 years	97	27.3
	More than 10 years	67	18.9
Sector	Financial services	82	23.1
	Technology	101	28.5
	Manufacturing	96	27.0
	Education	31	8.7
	Healthcare	24	6.8
	Other services	21	5.9

*N* = 355. Percentages may not total 100 because of rounding. Other services included retail, professional services, and other service industries.

### 3.2 Measures

All constructs were assessed using multi-item measures drawn from prior studies. Responses were recorded on a five-point Likert scale ranging from 1 (“strongly disagree”) to 5 (“strongly agree”).

Before data collection, the questionnaire was translated and back-translated. Two bilingual researchers independently translated the English items into Chinese and resolved discrepancies through discussion. A third bilingual translator subsequently back-translated the Chinese version into English (Brislin, 1970). The translation process emphasized conceptual equivalence and readability in the Chinese workplace context. The wording of the administered items and details of the scale adaptations are reported in Appendix A.

Before the formal survey, the Chinese-language questionnaire was pilot-tested with 30 employees who met the same eligibility criteria as the main-study participants. The pilot participants completed the questionnaire and provided feedback on item clarity, potentially ambiguous wording, contextual appropriateness, and the overall response process. Based on this feedback, minor adjustments were made to several Chinese expressions to improve readability and contextual fit. These revisions did not alter the substantive meaning of the items, the number of items, the construct structure, or the five-point response format. Preliminary reliability analysis indicated acceptable internal consistency, with Cronbach’s alpha values ranging from 0.76 to 0.89. Given the small pilot sample, these estimates were treated as an initial screening check rather than as formal psychometric validation. The pilot responses were used solely to refine the questionnaire and were not included in the final analytical sample.

Workplace spirituality was assessed using a study-specific abbreviated and adapted nine-item measure based on the three-dimensional operationalization reported by Milliman et al. (2003). Before translation and formal data collection, the research team selected three items from each dimension based on conceptual representativeness, avoidance of closely repetitive wording, balanced coverage of the three dimensions, and anticipated clarity and applicability in the Chinese workplace context. The remaining source items were not administered. The nine-item structure was specified before formal data collection and was not developed through *post hoc* item deletion based on the main-sample results. This balanced structure reduced respondent burden while retaining representation of meaningful work, sense of community, and alignment with organizational values. The pilot test did not result in any change to the number of items or the dimensional structure of the measure. Only minor adjustments were made to the Chinese wording to improve readability and contextual appropriateness. All nine administered workplace-spirituality items were retained in the final measurement model; no items were subsequently removed on the basis of factor loadings, reliability estimates, or structural-model results from the main sample. The wording of all nine workplace-spirituality items administered in the study is reported in Appendix A, and the complete Chinese-language questionnaire used for data collection is provided in Supplementary material S1.

Leader bottom-line mentality was measured using the four-item scale developed by Greenbaum et al. (2012). Innovative work behavior was measured using Janssen’s (2000) nine-item scale, which covers idea generation, idea promotion, and idea implementation. Individual–organizational value congruence was measured using three items adapted from Cable and DeRue (2002). Demographic information was also collected, including gender, age, education, organizational tenure, organizational position, and sector affiliation. Blue attitude was assessed as a theoretically unrelated marker variable using three items adapted from Miller and Simmering (2023).

### 3.3 Data analysis

The data were analyzed using partial least squares structural equation modeling (PLS-SEM) in SmartPLS 4 (Ringle et al., 2024). PLS-SEM was selected because the model includes multiple latent constructs, a reflective-reflective higher-order construct of workplace spirituality, direct and indirect associations, and a moderation effect (Hair et al., 2021; Henseler et al., 2016). Although covariance-based SEM can also estimate models of this type, PLS-SEM was adopted as a variance-based approach that allowed the latent-variable model, higher-order construct, and interaction term to be estimated within a single analytical framework (Hair et al., 2021).

The final analytical sample comprised 355 valid responses. Bootstrapping with 5,000 resamples was used to obtain confidence intervals and significance tests for the direct, indirect, total, and interaction effects (Hair et al., 2021). The ability to estimate and bootstrap the model was not treated as evidence of sample-size adequacy. Because no formal prospective power analysis was conducted for the smallest effect of interest, the non-significant interaction result was interpreted cautiously.

The analysis proceeded in two stages. First, the measurement model was assessed using indicator loadings, Cronbach’s alpha, composite reliability, and average variance extracted (Hair et al., 2021). Workplace spirituality was specified as a reflective-reflective higher-order construct in accordance with the conceptualization explained in Section 2.1.2. The higher-order construct was treated as an overarching work experience reflected in meaningful work, sense of community, and alignment with organizational values, rather than as a composite formed by three independent components. A two-stage approach was applied. In the first stage, latent-variable scores were estimated for the three reflective first-order constructs. In the second stage, the scores for meaningful work, sense of community, and alignment with organizational values were used as reflective indicators of the higher-order WS construct (Sarstedt et al., 2019; Hair et al., 2021). Discriminant validity was evaluated using the heterotrait–monotrait ratio of correlations (HTMT) (Henseler et al., 2015). Bootstrapped confidence intervals were also examined to determine whether the HTMT confidence intervals included 1. Because individual–organizational value congruence is conceptually close to the alignment-with-organizational-values dimension of workplace spirituality, an additional first-order construct-level HTMT assessment was conducted for AOV and IOVC (Henseler et al., 2016).

Second, the structural model was assessed using path coefficients, bootstrapped confidence intervals, *R*^2^, *f*^2^, and variance inflation factors (Hair et al., 2021). The hypothesized indirect association between leader BLM and IWB through WS was assessed using the bootstrapped indirect effect. The moderating role of individual–organizational value congruence was examined using the two-stage approach to estimate the interaction term between leader BLM and IOVC (Hair et al., 2021). Because organization- and supervisor-level identifiers were unavailable in the analytical dataset, potential clustering at these levels could not be tested or modeled. The structural analyses therefore focused on associations at the individual respondent level.

To examine whether the principal structural pattern was also evident at the dimension level, three separate first-order sensitivity models were estimated. Meaningful work, sense of community, and alignment with organizational values were examined individually as focal work-experience constructs. In each model, leader BLM was specified as a predictor of the focal dimension, while the focal dimension and leader BLM were specified as predictors of IWB. The corresponding indirect and total associations were estimated using bootstrapping. These analyses were conducted as structural sensitivity checks and were not used to validate the reflective-reflective higher-order measurement specification.

A second set of sensitivity analyses addressed the more specific concern regarding item-content overlap between IOVC and AOV. Two additional models were estimated after omitting AOV, with meaningful work and sense of community examined separately as the focal work-experience construct. In each model, leader BLM, IOVC, and their interaction term were specified as predictors of the focal construct, while the focal construct and leader BLM were specified as predictors of IWB. The corresponding indirect association between leader BLM and IWB through the focal construct was also estimated. These analyses assessed whether the principal findings remained evident when the overlapping AOV dimension was excluded. They were not treated as replacements for the primary three-dimensional conceptualization of WS.

Because all variables were collected from the same respondents at a single point in time, common method bias and causal ordering could not be ruled out completely. Procedural remedies included anonymity, voluntary participation, clear instructions, and separation of the construct sections within the questionnaire (Podsakoff et al., 2003; Podsakoff et al., 2012). Blue attitude, a theoretically unrelated marker variable, was used as a statistical diagnostic check for common method bias (Miller and Simmering, 2023).

## 4 Data analysis

### 4.1 Descriptive statistics and correlations

Table 2 presents the means, standard deviations, and Pearson correlations among the focal variables based on observed mean-composite scores. Leader BLM was negatively correlated with WS (*r* = −0.349, *p* < 0.001) and IWB (*r* = −0.149, *p* = 0.005). WS was positively correlated with IWB (*r* = 0.351, *p* < 0.001) and IOVC (*r* = 0.326, *p* < 0.001). IOVC was also positively correlated with IWB (*r* = 0.140, *p* = 0.008). These bivariate correlations were generally consistent with the expected directions. The correlations reported in Table 2 should be distinguished from the structural path estimates reported in Table 3.

**Table 2:** Means, standard deviations, and correlations of the study variables.

Variable	M	SD	1	2	3	4
Leader bottom-line mentality (BLM)	3.149	0.734	—			
Workplace spirituality (WS)	3.479	0.621	−0.349^**^	—		
Innovative work behavior (IWB)	3.550	0.660	−0.149^**^	0.351^**^	—	
Individual–organizational value congruence (IOVC)	3.379	0.640	−0.131^*^	0.326^**^	0.140^**^	—

*N* = 355. Means, standard deviations, and Pearson correlations were calculated using observed mean-composite scores. BLM, IWB, and IOVC were calculated as the means of their respective items, and WS was calculated as the mean of its nine administered items. These descriptive composite-score correlations are distinct from the latent-variable estimates used in the PLS-SEM analysis. *p < 0.05; **p < 0.01.

**Table 3:** Structural path and hypothesis testing results.

Hypothesis	Relationship	Effect type	*β*	*t*	*p*	95% CI	Result
H1	BLM → IWB	Direct	−0.030	0.518	0.605	[−0.145, 0.080]	Not supported
H2	BLM → WS	Direct	−0.312	6.549	<0.001	[−0.405, −0.219]	Supported
—	IOVC → WS	Constituent main effect	0.287	5.774	<0.001	[0.185, 0.381]	—
H3	WS → IWB	Direct	0.341	6.497	< 0.001	[0.234, 0.438]	Supported
H4	BLM → WS → IWB	Indirect	−0.106	4.583	<0.001	[−0.155, −0.063]	Supported
—	BLM → IWB	Total	−0.136	2.606	0.009	[−0.238, −0.034]	—
H5	BLM × IOVC → WS	Interaction	−0.030	0.638	0.524	[−0.122, 0.062]	Not supported

Bootstrapping with 5,000 resamples was used to estimate the significance of the direct, indirect, total, and interaction effects. The reported t statistics are absolute values, whereas the direction of each association is indicated by the sign of the path coefficient (*β*). Two-tailed significance tests were applied. CI = confidence interval; BLM = leader bottom-line mentality; WS = workplace spirituality; IWB = innovative work behavior; IOVC = individual–organizational value congruence. The IOVC → WS path was included as a constituent main effect in the moderation model and was not specified as a separate hypothesis.

### 4.2 Assessment of the measurement model

Following the analytical procedure described in Section 3.3, the measurement model was first assessed before testing the structural relationships. The assessment focused on indicator reliability, internal consistency reliability, convergent validity, and discriminant validity. Because WS was modeled as a reflective-reflective higher-order construct, the first-order constructs were assessed first, followed by the higher-order construct assessment.

#### 4.2.1 First-order measurement model

All first-order constructs were specified as reflective constructs. Indicator reliability was assessed using standardized factor loadings. Internal consistency reliability was evaluated using Cronbach’s alpha and composite reliability, and convergent validity was assessed using average variance extracted.

As shown in Table 4, the standardized loadings ranged from 0.698 to 0.886. All loadings were at or very close to the commonly used threshold of 0.70, with the lowest loading rounding to 0.70 at two decimal places. Cronbach’s alpha values ranged from 0.816 to 0.918, and composite reliability values ranged from 0.891 to 0.932, indicating satisfactory internal consistency reliability. The average variance extracted values ranged from 0.606 to 0.755, exceeding the recommended threshold of 0.50. These results support the indicator reliability, internal consistency reliability, and convergent validity of the first-order measurement model (Sarstedt et al., 2022).

**Table 4:** Measurement model assessment (first-order constructs).

Construct	Loading range	Cronbach’s *α*	CR	AVE
BLM	0.819–0.853	0.857	0.904	0.701
MW	0.849–0.885	0.831	0.899	0.748
SC	0.854–0.886	0.838	0.903	0.755
AOV	0.841–0.867	0.816	0.891	0.731
IWB	0.698–0.823	0.918	0.932	0.606
IOVC	0.854–0.870	0.824	0.895	0.740

#### 4.2.2 Second-order construct assessment

Workplace spirituality was specified as a reflective-reflective higher-order construct with meaningful work, sense of community, and alignment with organizational values as three reflective first-order dimensions. Under this specification, WS represents the common experiential orientation reflected in variation across the three dimensions. The dimensions were therefore modeled as manifestations of the higher-order construct rather than as independent components that combine to form WS (Sarstedt et al., 2019; Sarstedt et al., 2022).

Following the two-stage procedure described in Section 3.3, scores for meaningful work, sense of community, and alignment with organizational values were used as reflective indicators of the higher-order WS construct. As shown in Table 5, the loadings of meaningful work, sense of community, and alignment with organizational values were 0.857, 0.851, and 0.843, respectively. The similar and substantial loadings indicate that all three dimensions shared considerable variance with higher-order WS. The composite reliability of WS was 0.887, and the average variance extracted was 0.723, supporting the reliability and convergent validity of the higher-order specification (Hair et al., 2021). These results are consistent with the proposed reflective-reflective representation, although they do not establish that it is the only plausible measurement specification.

**Table 5:** Second-order construct assessment.

Second-order construct	First-order dimension	Loading
Workplace spirituality (WS)	Meaningful work (MW)	0.857
	Sense of community (SC)	0.851
	Alignment with organizational values (AOV)	0.843

CR = 0.887, AVE = 0.723. CR = composite reliability; AVE = average variance extracted.

#### 4.2.3 Discriminant validity

Discriminant validity was evaluated using the heterotrait–monotrait ratio of correlations (HTMT). As shown in Table 6, all HTMT ratios among the main constructs were below the conservative threshold of 0.85. Bootstrapped confidence intervals are reported in Appendix B, and none included 1, providing additional support for discriminant validity.

**Table 6:** HTMT ratios.

Construct	BLM	WS	IWB	IOVC
BLM	—			
WS	0.398	—		
IWB	0.169	0.387	—	
IOVC	0.156	0.380	0.163	—

HTMT = heterotrait–monotrait ratio. All HTMT values were below the recommended threshold of 0.85.

Because individual–organizational value congruence is conceptually close to the alignment-with-organizational-values dimension of workplace spirituality, an additional first-order construct-level HTMT assessment was conducted. The AOV–IOVC HTMT value was 0.462, and its bootstrapped 95% confidence interval [0.353, 0.568] did not include 1. These results indicate that AOV and IOVC were not statistically redundant under the HTMT criterion.

Nevertheless, statistical discriminant validity does not eliminate the substantive overlap evident in several item wordings. The results should therefore not be interpreted as demonstrating complete conceptual distinctiveness.

### 4.3 Common method bias

To provide a statistical diagnostic check, blue attitude was included as a theoretically unrelated marker variable. As reported in Appendix C, Table C1, its correlations with the focal constructs were small and non-significant, ranging from −0.025 to 0.048.

Blue attitude was subsequently included as an additional predictor of both endogenous constructs, WS and IWB. Its direct associations with WS (*β* = −0.031, *t* = 0.678, *p* = 0.498, 95% CI [−0.122, 0.059]) and IWB (*β* = 0.009, *t* = 0.174, *p* = 0.862, 95% CI [−0.087, 0.110]) were non-significant.

As shown in Appendix C, Table C2, inclusion of the marker variable did not substantively alter the focal structural results. The direction and significance conclusions remained unchanged for the BLM–WS association, the WS–IWB association, the direct BLM–IWB association, the BLM × IOVC interaction, and the indirect association between BLM and IWB through WS.

Taken together, the marker-variable analysis did not indicate substantial marker-related distortion of the focal associations. Nevertheless, common method bias cannot be ruled out completely because the study used a cross-sectional, single-source design.

### 4.4 Structural model assessment

The structural model was assessed using bootstrapping with 5,000 resamples. Before hypothesis testing, structural collinearity was examined using variance inflation factors. The VIF values ranged from 1.01 to 1.14, remaining well below the recommended threshold of 3.3. The interaction term between leader BLM and IOVC was estimated together with the two constituent main effects in the equation predicting WS.

As shown in Table 3, the direct association between leader BLM and IWB was negative but non-significant (*β* = −0.030, *t* = 0.518, *p* = 0.605, 95% CI [−0.145, 0.080]); therefore, H1 was not supported. Leader BLM was negatively associated with WS (*β* = −0.312, *t* = 6.549, *p* < 0.001, 95% CI [−0.405, −0.219]), supporting H2. WS was positively associated with IWB (*β* = 0.341, *t* = 6.497, *p* < 0.001, 95% CI [0.234, 0.438]), supporting H3.

The indirect association between leader BLM and IWB through WS was negative and significant (*β* = −0.106, *t* = 4.583, *p* < 0.001, 95% CI [−0.155, −0.063]), supporting H4. The total association between leader BLM and IWB was also negative and significant (*β* = −0.136, *t* = 2.606, *p* = 0.009, 95% CI [−0.238, −0.034]). Within the structural model, the negative total association comprised a significant negative indirect association through WS and a small, non-significant direct association.

IOVC was positively associated with WS as a constituent main effect in the moderation equation (*β* = 0.287, *t* = 5.774, *p* < 0.001, 95% CI [0.185, 0.381], *f*^2^ = 0.101, VIF = 1.02). This path was not specified as a separate hypothesis. The interaction between leader BLM and IOVC was not significant (*β* = −0.030, *t* = 0.638, *p* = 0.524, 95% CI [−0.122, 0.062]); therefore, H5 was not supported. This result indicates that IOVC did not significantly moderate the association between leader BLM and WS in the present sample.

The explanatory power of the structural model is reported in Table 7. The model explained 20.2% of the variance in WS (*R*^2^ = 0.202; adjusted *R*^2^ = 0.195) and 12.4% of the variance in IWB (*R*^2^ = 0.124; adjusted *R*^2^ = 0.119).

**Table 7:** Model explanatory power.

Endogenous construct	*R* ^2^	Adjusted *R*^2^
Workplace spirituality	0.202	0.195
Innovative work behavior	0.124	0.119

*R*^2^ represents the proportion of variance explained in each endogenous construct. Adjusted *R*^2^ accounts for the number of predictors relative to the sample size.

Effect sizes were assessed using *f*^2^. As shown in Table 8, the effects of leader BLM on WS (*f*^2^ = 0.120), WS on IWB (*f*^2^ = 0.116), and IOVC on WS (*f*^2^ = 0.101) were small in magnitude. In contrast, the direct effect of leader BLM on IWB (*f*^2^ = 0.001) and the interaction effect of leader BLM and IOVC on WS (*f*^2^ = 0.001) were negligible.

**Table 8:** Effect size (*f*^2^).

Path	*f* ^2^	Effect size
BLM → WS	0.120	Small
WS → IWB	0.116	Small
IOVC → WS	0.101	Small
BLM → IWB	0.001	Negligible
BLM × IOVC → WS	0.001	Negligible

Following commonly used guidelines, *f*^2^ values of 0.02, 0.15, and 0.35 indicate small, medium, and large effect sizes, respectively. Values below 0.02 were interpreted as negligible.

### 4.5 Robustness and additional checks

The structural model was re-estimated with gender, age, education, organizational tenure, and position as controls. The focal results remained substantively unchanged. Leader BLM remained negatively associated with WS (*β* = −0.306, *p* < 0.001), WS remained positively associated with IWB (*β* = 0.340, *p* < 0.001), and the indirect association through WS remained negative and significant (*β* = −0.104, *p* < 0.001). The direct BLM–IWB association and the BLM × IOVC interaction remained non-significant (Appendix Table D1).

The reverse-path model produced a significant indirect association from IWB to BLM through WS (*β* = −0.119, *p* < 0.001), whereas the direct IWB–BLM association was non-significant (*β* = −0.030, *p* = 0.606). Given the cross-sectional design, this result was treated only as a sensitivity check and does not establish temporal or causal ordering (Appendix Table D2).

The dimension-specific models were estimated separately. Leader BLM was negatively associated with meaningful work, sense of community, and alignment with organizational values, while each dimension was positively associated with IWB. The indirect associations were significant through meaningful work (*β* = −0.080, *p* < 0.001), sense of community (*β* = −0.093, *p* < 0.001), and alignment with organizational values (*β* = −0.076, *p* < 0.001). Because the same sample and the same BLM and IWB scores were used, the common total BLM–IWB association is reported once in Appendix Table D3.

The two AOV-omitted specifications were also estimated separately. In the meaningful-work model, the BLM × IOVC interaction was non-significant (*β* = 0.029, *p* = 0.571), while the indirect association through meaningful work remained negative and significant (*β* = −0.073, *p* < 0.001). In the sense-of-community model, the interaction was likewise non-significant (*β* = −0.003, *p* = 0.949), while the indirect association through sense of community remained negative and significant (*β* = −0.085, *p* < 0.001). These results indicate that the negative indirect pattern remained stable after excluding AOV, whereas the proposed moderating role of IOVC was not supported (Appendix Table D4).

## 5 Discussion

### 5.1 Summary of findings

The results most clearly supported a negative indirect association between leader BLM and IWB through workplace spirituality. Although leader BLM was negatively correlated with IWB at the observed composite-score level, its direct association with IWB was small and non-significant in the latent-variable structural model. By contrast, the indirect association through workplace spirituality was negative and significant, indicating that the overall negative association was primarily reflected in the indirect path rather than in a direct relationship.

Decomposing the indirect association, leader BLM was negatively associated with workplace spirituality, whereas workplace spirituality was positively associated with IWB. Employees who perceived a stronger bottom-line emphasis reported lower levels of meaningful work, sense of community, and alignment with organizational values, while employees reporting stronger workplace spirituality also reported greater innovative work behavior. This pattern identifies workplace spirituality as the work-experience component most clearly associated with the observed BLM–IWB relationship, without establishing temporal or causal ordering.

The proposed moderating role of IOVC was not supported. The interaction between leader BLM and IOVC was non-significant, indicating that perceived value congruence did not significantly weaken the negative BLM–WS association in this sample. This conclusion remained unchanged in the sensitivity models excluding alignment with organizational values. Thus, the absence of moderation was not confined to the primary specification of workplace spirituality, although the result should be interpreted as a lack of supporting evidence rather than definitive evidence that no moderating effect exists.

### 5.2 Theoretical implications

The study contributes to BLM research by reframing employee innovation as more than a direct behavioral correlate of bottom-line-oriented leadership. Existing studies have primarily examined unethical conduct, interpersonal strain, silence, and performance-related responses (Babalola et al., 2020; Eissa et al., 2020; Greenbaum et al., 2023). By examining IWB, the present study extends this literature to a discretionary and uncertain form of behavior whose value is not always immediately visible. The findings therefore qualify a simple direct-association account and indicate that the BLM–IWB relationship is more appropriately interpreted in conjunction with employees’ reported experience of work.

A more specific contribution lies in positioning workplace spirituality as an integrated work-experience construct encompassing meaningful work, sense of community, and alignment with organizational values (Milliman et al., 2003). This perspective differs from treating WS as merely another favorable workplace condition: it brings together employees’ sense of purpose, social connection, and value consistency, all of which are relevant to discretionary innovative effort. The significant indirect association is thus consistent with WS as a theoretically relevant lens through which the observed BLM–IWB relationship can be understood, complementing accounts that emphasize risk, performance pressure, or idea-related processes (Greenbaum et al., 2020; Luo et al., 2025; Sode and Chenji, 2024). Because the design is cross-sectional, this contribution concerns the interpretation of the association rather than evidence of a causal mechanism.

The non-significant IOVC interaction also refines the proposed boundary condition. Person–organization fit research suggests that value congruence may shape how employees interpret organizational demands (Kristof, 1996; Cable and DeRue, 2002; Edwards and Cable, 2009), but the present findings provided no evidence that general value fit buffered the negative BLM–WS association. This result cautions against assuming that perceived compatibility with organizational values is sufficient to offset a narrowly communicated bottom-line orientation. It also highlights the need to distinguish general value congruence more clearly from the alignment-with-organizational-values dimension of WS. Although the AOV-omitted sensitivity analyses yielded the same non-significant interaction pattern, the conceptual proximity of the two constructs remains important for future measurement and theory development.

### 5.3 Practical implications

For organizations, the findings point to the importance of balancing clear performance expectations with working conditions that leave room for employee innovation. The results do not suggest that bottom-line goals are inherently harmful (Greenbaum et al., 2023; Metwally and Abdelmotaleb, 2025). Rather, the practical concern arises when leaders communicate measurable outcomes as the only priorities that matter, which may cause learning, collaboration, ethical conduct, and long-term improvement to appear secondary.

Managers can support a broader work experience by explaining why performance targets matter, connecting employees’ tasks with the wider purpose of the organization, and recognizing contributions whose value may not be immediately reflected in numerical indicators. They may also encourage idea sharing, maintain respectful workplace relationships, and allow reasonable space for experimentation and learning from unsuccessful attempts. Such practices may help keep meaningful work, community, and organizational values visible alongside performance demands, thereby preserving conditions in which employees are more willing to develop and advance new ideas (Alfarajat and Emeagwali, 2021; Hunsaker and Ding, 2022; Kim and Song, 2024).

The non-significant IOVC interaction also calls for practical caution. The findings provide no evidence that recruiting or socializing employees for general value fit is, by itself, sufficient to weaken the negative association between leader BLM and workplace spirituality. Organizations may therefore need to look beyond broad statements of value congruence and pay closer attention to everyday managerial practices, including how goals are explained, how mistakes are handled, and how employee ideas are evaluated. Because the study used cross-sectional, single-source data, these recommendations should be interpreted as context-dependent practical suggestions rather than causally validated interventions.

### 5.4 Limitations and future research

Several limitations should be considered when interpreting the findings. First, the cross-sectional design does not establish the temporal or causal ordering among leader BLM, workplace spirituality, and IWB. Although the hypothesized model was theoretically grounded, the data cannot determine whether leader BLM precedes changes in employees’ work experience and innovative behavior. Future research could use longitudinal, time-lagged, or experimental designs to examine the proposed sequence more directly.

Second, all substantive variables were reported by the same respondents. The procedural remedies and marker-variable analyses reduced some concerns, but they cannot completely rule out common method bias. Future studies could combine employee reports of leader BLM and workplace spirituality with supervisor assessments of IWB or objective indicators of innovation-related contributions. Such multi-source designs would provide evidence beyond employees’ perceptions and reduce reliance on a single measurement source.

Third, the sampling context and data structure limit the generalizability and level of analysis of the findings. The participants were employees aged 18–43 working in Henan Province, China, and the observed relationships may not generalize to other regions, age groups, industries, or cultural settings. Moreover, because the anonymous dataset did not retain organization- or supervisor-level identifiers, possible clustering could not be verified or modeled. Future research should examine more diverse samples, retain anonymous nesting codes, and collect data from multiple employees within supervisors, teams, and organizations to permit multilevel analysis.

Fourth, the study did not conduct an *a priori* sample-size calculation based on the smallest interaction effect of interest. The non-significant IOVC interaction should therefore be interpreted as an absence of supporting evidence in the present sample rather than definitive evidence that no moderating effect exists. Future studies should use effect-specific power analysis and larger samples where necessary to improve sensitivity to potentially small interaction effects.

Finally, the measurement of workplace spirituality requires further validation. The study used an abbreviated and adapted nine-item measure, with three items representing each first-order dimension. Although translation, back-translation, and pilot testing supported linguistic clarity, these procedures do not constitute comprehensive psychometric validation of the abbreviated Chinese measure. Future research could compare the abbreviated measure with the longer operationalization, conduct cognitive interviews and expert content assessments, and validate the measure in independent samples. It could also compare the reflective-reflective higher-order specification with reflective-formative and correlated first-order alternatives. In addition, future measures should distinguish IOVC more clearly from the alignment-with-organizational-values dimension of workplace spirituality and examine whether motivational constructs provide explanatory value beyond meaning, community, and value alignment.

## 6 Conclusion

This study examined the relationship between leader bottom-line mentality and employees’ innovative work behavior by considering workplace spirituality and individual–organizational value congruence. The findings were more consistent with a negative indirect association through employees’ reported experience of meaningful work, community, and value alignment than with a uniform direct relationship between leader BLM and IWB. The proposed buffering role of IOVC was not supported, suggesting that general value fit alone may not be sufficient to offset the work-experience implications associated with a strong bottom-line emphasis. Overall, the study positions workplace spirituality as an important consideration in understanding how bottom-line-oriented leadership relates to employee innovation. Organizations seeking performance discipline and innovation simultaneously should therefore remain attentive to whether employees continue to experience purpose, connection, and broader value consistency at work. Given the cross-sectional and single-source design, these conclusions should be understood as associational and require further examination through longitudinal and multi-source research.

## Data Availability

The raw data supporting the conclusions of this article will be made available by the authors, without undue reservation.
