# Supplementary material for: Leader bottom-line mentality and employees’ innovative work behavior: the role of workplace spirituality and value congruence
Source: Front Psychol. 2026 Sep 1;17:1912519. doi: 10.3389/fpsyg.2026.1912519 (PMC13574575; doi:10.3389/fpsyg.2026.1912519)
Supplement: Supplementary file 1 [file Data_Sheet_1.pdf]

Supplementary Material S1

正式调查所使用的中文问卷

Chinese-Language Questionnaire Administered in the Main Survey

研究说明与知情同意

尊敬的参与者：

您好！本问卷旨在了解领导者工作重点、员工工作体验、个人与组织价值观一致性以及员工创新工作行为之间的关系。调查结果仅用于学术研究。

您的参与完全自愿。问卷采用匿名方式，不要求提供姓名、单位名称或其他可直接识别个人身份的信息。问卷不存在正确或错误答案，请根据您的真实经历和感受作答。在提交问卷之前，您可以随时停止作答并退出本研究。

参加本研究的基本条件为：年龄在 18 至 43 岁之间；目前在河南省的组织中工作；已在现组织工作至少 6 个月；并有一位直接主管。管理人员或高层管理人员在回答有关主管的问题时，请评价您自己的直接主管，而不是评价您本人。

选择"我已阅读上述说明并自愿同意参加本研究"并继续作答，即表示您已知情并同意参加本研究。

☐	我已阅读上述说明并自愿同意参加本研究。
☐	我不同意参加本研究（问卷结束）。

参与资格筛选

序号	筛选题	选项
1	您的年龄是否在 18 至 43 岁之间？	☐是 ☐否
2	您目前是否在河南省的组织中工作？	☐是 ☐否
3	您在当前组织的工作时间是否已满 6 个月？	☐是 ☐否
4	您目前是否有一位直接主管？	☐是 ☐否

上述筛选信息用于确认参与资格，并与问卷后面的人口统计信息进行一致性核对，主要包括年龄和在当前组织的工作年限。

量表题项

请根据您的真实情况，选择最符合您看法的选项。所有题项均采用 5 点量表：

1 = 非常不同意　2 = 不同意　3 = 一般　4 = 同意　5 = 非常同意

1、关于您的直接主管

请根据您对直属主管日常工作重点的真实感受作答。管理人员或高层管理人员应评价自己的直接主管。

序号	题 项	1	2	3	4	5
BLM1	我的直接主管将底线结果视为最重要的工作事项。	☐	☐	☐	☐	☐
BLM2	我的直接主管主要关注可衡量的绩效结果。	☐	☐	☐	☐	☐
BLM3	我的直接主管将底线结果置于其他工作事项之上。	☐	☐	☐	☐	☐
BLM4	我的直接主管在作出与工作有关的决策时，会优先考虑绩效目标。	☐	☐	☐	☐	☐

2、关于您的工作意义体验

请根据您对目前工作的实际感受作答。

序号	题 项	1	2	3	4	5
MW1	我认为自己的工作是有意义的。	☐	☐	☐	☐	☐
MW2	我的工作与我认为人生中重要的事情相联系。	☐	☐	☐	☐	☐
MW3	我感到自己的工作具有明确而有意义的目的。	☐	☐	☐	☐	☐

3、关于您的工作关系体验

请根据您对工作场所人际关系的实际感受作答。

序号	题 项	1	2	3	4	5
SC1	我觉得自己是工作共同体的一员。	☐	☐	☐	☐	☐
SC2	我所在工作场所的人们相互支持。	☐	☐	☐	☐	☐
SC3	我与一起工作的同事之间有联结感。	☐	☐	☐	☐	☐

4、关于您与组织使命及价值观的关系

请根据您对所在组织使命与价值观的实际感受作答。

序号	题 项	1	2	3	4	5
AOV1	我认同所在组织的价值观。	☐	☐	☐	☐	☐
AOV2	我所在组织的价值观与我个人的价值观一致。	☐	☐	☐	☐	☐
AOV3	我对所在组织所倡导的使命和价值理念具有认同感。	☐	☐	☐	☐	☐

5、关于您的创新工作行为

请根据您在目前工作中的实际行为作答。

序号	题 项	1	2	3	4	5
IWB1	我会提出新的想法来解决工作中的问题。	☐	☐	☐	☐	☐
IWB2	我会在工作中寻找新的方法、技术或途径。	☐	☐	☐	☐	☐
IWB3	我会针对工作中的问题提出原创性的解决方案。	☐	☐	☐	☐	☐
IWB4	我会向工作中的其他人积极推广新想法。	☐	☐	☐	☐	☐
IWB5	我会努力为创新想法争取支持。	☐	☐	☐	☐	☐
IWB6	我会努力让组织中的关键人员对创新想法产生兴趣。	☐	☐	☐	☐	☐

IWB7	我会将创新想法转化为实际应用。	☐	☐	☐	☐	☐
IWB8	我会把创新想法引入自己的工作。	☐	☐	☐	☐	☐
IWB9	我会评估创新想法在实践中是否有用。	☐	☐	☐	☐	☐

6、关于您与所在组织的价值观一致性

请根据您的个人价值观与组织价值观的实际匹配程度作答。

序号	题 项	1	2	3	4	5
IOVC1	我的个人价值观与所在组织的价值观相匹配。	☐	☐	☐	☐	☐
IOVC2	我在生活中重视的事物与所在组织所重视的事物相似。	☐	☐	☐	☐	☐
IOVC3	我与所在组织在核心价值取向上十分契合。	☐	☐	☐	☐	☐

7、关于颜色偏好

请根据您的真实感受作答。

序号	题 项	1	2	3	4	5
BA1	我喜欢蓝色。	☐	☐	☐	☐	☐
BA2	我觉得蓝色令人愉悦。	☐	☐	☐	☐	☐
BA3	我对蓝色持积极态度。	☐	☐	☐	☐	☐

人口统计信息

请根据您的实际情况选择相应选项。以下信息仅用于描述样本整体特征，不用于识别个人身份。

序号	问题	选项
1	您的性别	☐ 男性；<br>☐ 女性
2	您的年龄	☐ 18 岁以下；<br>☐ 18—23 岁；<br>☐ 24—33 岁；<br>☐ 34—43 岁；<br>☐ 44 岁及以上
3	您的最高受教育程度	☐ 中学学历；<br>☐ 大专 / 职业技术学校学历；<br>☐ 学士学位；<br>☐ 硕士学位
4	您目前的职位	☐ 非管理人员；<br>☐ 管理人员；<br>☐ 高层管理人员
5	您在当前组织的工作年限	☐ 不足 6 个月；<br>☐ 6 个月至 1 年；<br>☐ 1—5 年；<br>☐ 6—10 年；<br>☐ 10 年以上
6	您所在组织主要属于以下哪个行业？	☐ 金融服务业<br>☐ 科技行业<br>☐ 制造业<br>☐ 教育行业<br>☐ 医疗健康行业<br>☐ 其他服务业
