# Supplementary material for: Leader bottom-line mentality and employees’ innovative work behavior: the role of workplace spirituality and value congruence
Source: Front Psychol. 2026 Sep 1;17:1912519. doi: 10.3389/fpsyg.2026.1912519 (PMC13574575; doi:10.3389/fpsyg.2026.1912519)
Supplement: Supplementary file 2 [file Data_Sheet_2.pdf]

Supplementary Material S1

English Translation of the Chinese-Language Questionnaire Administered in the Main Survey

Study Information and Informed Consent

Dear Participant:

This questionnaire aims to understand the relationships among leaders' work priorities, employees' work experiences, individual-organizational value congruence, and employees' innovative work behavior. The survey results will be used solely for academic research.

Your participation is entirely voluntary. The questionnaire is anonymous and does not ask for your name, organization name, or other information that can directly identify you. There are no right or wrong answers. Please respond based on your actual experiences and feelings. Before submitting the questionnaire, you may stop responding and withdraw from the study at any time.

To participate in this study, you must be 18-43 years old, currently work in an organization in Henan Province, have worked in your current organization for at least 6 months, and have an immediate supervisor. Managers and executives should evaluate their own immediate supervisor when answering supervisor-related questions, rather than evaluating themselves.

By selecting "I have read the information above and voluntarily agree to participate in this study" and continuing with the questionnaire, you indicate that you have been informed about the study and consent to participate.

Choice	Response
1	I have read the information above and voluntarily agree to participate in this study.
2	I do not agree to participate in this study (questionnaire ends).

Eligibility Screening

No.	Screening question	Options
1	Are you between 18 and 43 years old?	Yes / No
2	Do you currently work in an organization in Henan Province?	Yes / No
3	Have you worked in your current organization for at least 6 months?	Yes / No
4	Do you currently have an immediate supervisor?	Yes / No

The screening information above was used to confirm eligibility and to check consistency with the demographic information later in the questionnaire, particularly age and organizational tenure.

Measurement Items

Please select the option that best reflects your view based on your actual situation. All items use a five-point scale:

1 = Strongly disagree 2 = Disagree 3 = Neither agree nor disagree 4 = Agree 5 = Strongly agree

1. About Your Immediate Supervisor

Please respond based on your actual perceptions of your immediate supervisor's day-to-day work priorities. Managers and executives should evaluate their own immediate supervisor.

Code	Item	1	2	3	4	5
BLM1	My immediate supervisor treats bottom-line outcomes as the most important work priority.					
BLM2	My immediate supervisor focuses mainly on achieving measurable performance results.					
BLM3	My immediate supervisor places bottom-line outcomes above other workplace concerns.					
BLM4	My immediate supervisor gives priority to performance targets when making work-related decisions.					

2. About Your Experience of Meaningful Work

Please respond based on your actual experience of your current work.

Code	Item	1	2	3	4	5
MW1	I experience my work as meaningful.					
MW2	My work is connected to what I consider important in life.					
MW3	I feel that my work has a meaningful purpose.					

3. About Your Workplace Relationship Experience

Please respond based on your actual experience of interpersonal relationships in your workplace.

Code	Item	1	2	3	4	5
SC1	I feel part of a community at work.					
SC2	People in my workplace support one another.					
SC3	I feel connected with the people I work with.					

4. About Your Relationship With the Organization's Mission and Values

Please respond based on your actual experience of your organization's mission and values.

Code	Item	1	2	3	4	5
AOV1	I feel aligned with the values of my organization.					
AOV2	My organization's values are consistent with my own values.					
AOV3	I identify with the mission and value principles promoted by my organization.					

Measurement Items (continued)

5. About Your Innovative Work Behavior

Please respond based on your actual behavior in your current work.

Code	Item	1	2	3	4	5
IWB1	I create new ideas to solve work-related problems.					
IWB2	I search for new methods, techniques, or approaches at work.					
IWB3	I generate original solutions to work-related issues.					
IWB4	I promote new ideas to others at work.					
IWB5	I try to obtain support for innovative ideas.					
IWB6	I make important people in the organization enthusiastic about innovative ideas.					
IWB7	I transform innovative ideas into practical applications.					
IWB8	I introduce innovative ideas into my work.					
IWB9	I evaluate whether innovative ideas can be useful in practice.					

6. About Value Congruence Between You and Your Organization

Please respond based on the actual degree of fit between your personal values and your organization's values.

Code	Item	1	2	3	4	5
IOVC1	My personal values match the values of my organization.					
IOVC2	The things I value in life are similar to the things my organization values.					
IOVC3	My core value orientation fits well with that of my organization.					

7. About Color Preference

Please respond based on your actual feelings.

Code	Item	1	2	3	4	5
BA1	I like the color blue.					
BA2	The color blue is pleasant to me.					
BA3	I have a favorable attitude toward the color blue.					

Note. This document is an English translation prepared for production. The main survey was administered in Chinese. The English wording of all measurement items corresponds to the administered Chinese items and the final measurement-item wording reported in the Supplementary Appendix.

Demographic Information

Please select the option that corresponds to your actual situation. The following information is used only to describe the overall characteristics of the sample and not to identify individual participants.

No.	Question	Options
1	Gender	Male<br>Female
2	Age	Under 18<br>18-23 years<br>24-33 years<br>34-43 years<br>44 years or older
3	Highest level of education	Secondary school certificate<br>Diploma / technical school certificate<br>Bachelor's degree<br>Master's degree
4	Current position	Non-managerial employee<br>Manager<br>Executive
5	Tenure in current organization	Less than 6 months<br>6 months to 1 year<br>1-5 years<br>6-10 years<br>More than 10 years
6	Which of the following best describes the main industry of your organization?	Financial services<br>Technology<br>Manufacturing<br>Education<br>Healthcare<br>Other services

Note. The demographic response options mirror those in the Chinese-language questionnaire used for data collection.
