# Supplementary material for: Leader bottom-line mentality and employees’ innovative work behavior: the role of workplace spirituality and value congruence
Source: Front Psychol. 2026 Sep 1;17:1912519. doi: 10.3389/fpsyg.2026.1912519 (PMC13574575; doi:10.3389/fpsyg.2026.1912519)
Supplement: Supplementary file 3 [file Supplementary_file_1.pdf]

Appendix A

Measurement items administered in the main survey

Construct	Code	English wording corresponding to the administered item	Source
Leader bottom-line mentality	BLM1	My immediate supervisor treats bottom-line outcomes as the most important work priority.	Greenbaum et al. (2012)
	BLM2	My immediate supervisor focuses mainly on achieving measurable performance results.	
	BLM3	My immediate supervisor places bottom-line outcomes above other workplace concerns.	
	BLM4	My immediate supervisor gives priority to performance targets when making work-related decisions.	
Meaningful work	MW1	I experience my work as meaningful.	Based on Milliman et al. (2003); abbreviated and adapted
	MW2	My work is connected to what I consider important in life.	
	MW3	I feel that my work has a meaningful purpose.	
Sense of community	SC1	I feel part of a community at work.	
	SC2	People in my workplace support one another.	
	SC3	I feel connected with the people I work with.	
Alignment with organizational values	AOV1	I feel aligned with the values of my organization.	
	AOV2	My organization's values are consistent with my own values.	
	AOV3	I identify with the mission and value principles promoted by my organization.	
Innovative work behavior	IWB1	I create new ideas to solve work-related problems.	Janssen (2000)
	IWB2	I search for new methods, techniques, or approaches at work.	
	IWB3	I generate original solutions to work-related issues.	
	IWB4	I promote new ideas to others at work.	
	IWB5	I try to obtain support for innovative ideas.	
	IWB6	I make important people in the organization enthusiastic about innovative ideas.	
	IWB7	I transform innovative ideas into practical applications.	
	IWB8	I introduce innovative ideas into my work.	
	IWB9	I evaluate whether innovative ideas can be useful in practice.	
Individual–organizational value congruence	IOVC1	My personal values match the values of my organization.	Cable and DeRue (2002)
	IOVC2	The things I value in life are similar to the things my organization values.	
	IOVC3	My core value orientation fits well with that of my organization.	
Blue attitude	BA1	I like the color blue.	Miller and Simmering (2023)
	BA2	The color blue is pleasant to me.	
	BA3	I have a favorable attitude toward the color blue.	

Note. All items were measured on a five-point Likert scale ranging from 1 = strongly disagree to 5 = strongly agree. The English wording reported in this appendix corresponds to the Chinese-language items administered to participants following the translation and back-translation procedure. Minor adjustments were made to several Chinese expressions during pilot testing to improve readability and contextual appropriateness, without changing the substantive meaning of the items, the number of items,

or the response format. The nine workplace-spirituality items reported here—MW1–MW3, SC1–SC3, and AOV1–AOV3—constitute the complete set of workplace-spirituality items administered in the main survey. The study used an abbreviated and adapted nine-item measure based on the three-dimensional operationalization reported by Milliman et al. (2003), rather than the complete item set reported in that study. The nine-item structure was specified before formal data collection, and all nine administered items were retained in the final measurement model. The complete Chinese-language questionnaire used for data collection is provided in Supplementary Material S1.

Appendix B

Bootstrapped confidence intervals for HTMT ratios

Construct pair	HTMT	2.5%	97.5%
BLM–WS	.398	.296	.499
BLM–IWB	.169	.096	.278
BLM–IOVC	.156	.077	.278
WS–IWB	.387	.285	.487
WS–IOVC	.380	.268	.486
IWB–IOVC	.163	.086	.280
AOV–IOVC	.462	.353	.568

Note. HTMT = heterotrait–monotrait ratio; CI = confidence interval; BLM = leader bottom-line mentality; WS = workplace spirituality; IWB = innovative work behavior; IOVC = individual–organizational value congruence; AOV = alignment with organizational values. The AOV–IOVC comparison was included because IOVC is conceptually close to the alignment-with-organizational-values dimension of workplace spirituality.

Appendix C

Marker-variable diagnostics for common method bias

Table C1

Correlations between blue attitude and focal constructs

Construct	Correlation with blue attitude	*p*
Leader BLM	.026	.626
WS	−.025	.633
IWB	−.001	.991
IOVC	.048	.369

Note. BLM = leader bottom-line mentality; WS = workplace spirituality; IWB = innovative work behavior; IOVC = individual–organizational value congruence.

Table C2

Comparison of structural results before and after including blue attitude

Path	Main model β	Main model *t*	Main model *p*	Marker-variable model β	Marker-variable model *t*	Marker-variable model *p*	Interpretation
BLM → WS	−.312	6.549	< .001	−.311	6.568	< .001	Substantively unchanged
WS → IWB	.341	6.497	< .001	.341	6.614	< .001	Substantively unchanged
BLM → IWB	−.030	.518	.605	−.030	.525	.600	Substantively unchanged
BLM × IOVC → WS	−.030	.638	.524	−.029	.628	.530	Substantively unchanged
BLM → WS → IWB	−.106	4.583	< .001	−.106	4.680	< .001	Substantively unchanged

Note. Blue attitude was included as a theoretically unrelated marker variable and modeled as an additional predictor of WS and IWB. The table compares the main structural model with the separately estimated marker-variable model. The reported *t* statistics are absolute values, and two-tailed significance tests were applied. “Substantively unchanged” indicates that the direction and significance conclusion of the focal association remained unchanged after inclusion of the marker variable. BLM = leader bottom-line mentality; WS = workplace spirituality; IWB = innovative work behavior; IOVC = individual–organizational value congruence.

Table C3

Effects of blue attitude on endogenous constructs

Path	β	*t*	*p*	95% CI
**Blue attitude → WS**	−.031	.678	.498	[−.122, .059]
**Blue attitude → IWB**	.009	.174	.862	[−.087, .110]

Note. The direct associations of blue attitude with WS and IWB were nonsignificant, and both confidence intervals included zero. These results indicate that the marker variable did not have a substantive association with either endogenous construct in the marker-variable model.

Appendix D

Robustness and additional checks

Table D1

Structural results with demographic controls

Path	Main model β	Main model *t*	Main model *p*	Control-variable model β	Control-variable model *t*	Control-variable model *p*	Interpretation
BLM → WS	−.312	6.549	< .001	−.306	6.192	< .001	Substantively unchanged
WS → IWB	.341	6.497	< .001	.340	6.454	< .001	Substantively unchanged
BLM → IWB	−.030	.518	.605	−.016	.281	.779	Substantively unchanged
BLM × IOVC → WS	−.030	.638	.524	−.025	.521	.602	Substantively unchanged
BLM → WS → IWB	−.106	4.583	< .001	−.104	4.405	< .001	Substantively unchanged

Note. The control-variable model included gender, age, education, organizational tenure, and position as additional predictors of WS and IWB. The main and control-variable models were estimated separately. The reported *t* statistics are absolute values, and two-tailed significance tests were applied. "Substantively unchanged" indicates that the direction and significance conclusion remained unchanged after inclusion of the controls. BLM = leader bottom-line mentality; WS = workplace spirituality; IWB = innovative work behavior; IOVC = individual–organizational value congruence.

Table D2

Alternative reverse-path model results

Panel A.

Path estimates

Relationship	Effect type	β	*t*	*p*	95% CI	VIF
IWB → WS	Direct	.351	7.714	< .001	[.257, .437]	1.00
WS → BLM	Direct	−.338	6.315	< .001	[−.441, −.229]	1.14
IWB → BLM	Direct	−.030	.516	.606	[−.143, .081]	1.14
IWB → WS → BLM	Indirect	−.119	4.814	< .001	[−.170, −.074]	—
IWB → BLM	Total	−.149	2.829	.005	[−.251, −.044]	—

Panel B.

Explained variance

Endogenous construct	R^2	Adjusted R^2
WS	.123	.121
BLM	.122	.117

Note. This alternative reverse-path model was estimated as a descriptive sensitivity check. Because the hypothesized and alternative specifications do not contain the same set of endogenous constructs, the R^2 values reported here should not be directly compared with those of the hypothesized model as evidence that either model has superior overall explanatory power. Bootstrapping with 5,000 resamples was used to estimate the significance of the direct, indirect, and total effects. The analysis does not establish temporal or causal ordering. β = standardized path coefficient; BLM = leader bottom-line mentality; WS = workplace spirituality; IWB = innovative work behavior; CI = confidence interval; VIF = variance inflation factor. VIF values are reported only for direct predictor relationships. Two-tailed tests were applied.

Table D3

Structural sensitivity analyses using the first-order dimensions of workplace spirituality

Panel A.

Meaningful-work model

Relationship	Effect type	β	*t*	*p*	95% CI
**BLM → MW**	Direct	−.274	5.245	< .001	[−.375, −.169]
**MW → IWB**	Direct	.292	6.210	< .001	[.198, .383]
**BLM → IWB**	Direct	−.069	1.218	.223	[−.177, .040]
**BLM → MW → IWB**	Indirect	−.080	3.861	< .001	[−.125, −.044]

Panel B.

Sense-of-community model

Relationship	Effect type	β	*t*	*p*	95% CI
**BLM → SC**	Direct	−.347	7.670	< .001	[−.435, −.258]
**SC → IWB**	Direct	.267	4.730	< .001	[.156, .377]
**BLM → IWB**	Direct	−.056	.935	.350	[−.172, .059]
**BLM → SC → IWB**	Indirect	−.093	4.001	< .001	[−.141, −.051]

Panel C.

Alignment-with-organizational-values model

Relationship	Effect type	β	*t*	*p*	95% CI
**BLM → AOV**	Direct	−.271	5.482	< .001	[−.371, −.175]
**AOV → IWB**	Direct	.280	5.112	< .001	[.168, .386]
**BLM → IWB**	Direct	−.073	1.325	.185	[−.179, .033]
**BLM → AOV → IWB**	Indirect	−.076	3.713	< .001	[−.120, −.040]
**BLM → IWB**	Total	−.149	2.829	.005	[−.251, −.044]

Panel D.

Explained variance

Model	Endogenous construct	R^2	Adjusted R^2
Meaningful-work model	MW	.075	.072
Meaningful-work model	IWB	.101	.096
Sense-of-community model	SC	.120	.118
Sense-of-community model	IWB	.085	.080
AOV model	AOV	.073	.071
AOV model	IWB	.095	.090

Note. Three separate dimension-specific models were estimated. In each model, leader BLM predicted one focal first-order dimension, while the focal dimension and leader BLM predicted IWB. Because the same sample and the same BLM and IWB scores were used across the three specifications, the models decomposed the same total BLM–IWB association into different direct and dimension-specific indirect components. The common total association was therefore reported once rather than repeated in each panel. Bootstrapping with 5,000 resamples was used, and two-tailed tests were applied. The reported t statistics are absolute values. BLM = leader bottom-line mentality; MW = meaningful work; SC = sense of community; AOV = alignment with organizational values; IWB = innovative work behavior; CI = confidence interval.

Table D4

Dimension-specific sensitivity analyses excluding AOV

Panel A.

Meaningful-work specification

Relationship	Effect type	β	*t*	*p*	95% CI
**BLM → MW**	Direct	−.250	4.792	< .001	[−.351, −.146]
**IOVC → MW**	Constituent main effect	.169	3.612	< .001	[.077, .260]
**BLM × IOVC → MW**	Interaction	.029	.567	.571	[−.071, .126]
**MW → IWB**	Direct	.292	6.210	< .001	[.198, .383]
**BLM → IWB**	Direct	−.069	1.218	.223	[−.177, .040]
**BLM → MW → IWB**	Indirect	−.073	3.656	< .001	[−.117, −.038]
**BLM → IWB**	Total	−.142	2.693	.007	[−.244, −.038]

Panel B.

Sense-of-community specification

Relationship	Effect type	β	*t*	*p*	95% CI
**BLM → SC**	Direct	−.320	6.673	< .001	[−.410, −.224]
**IOVC → SC**	Constituent main effect	.210	3.960	< .001	[.105, .314]
**BLM × IOVC → SC**	Interaction	−.003	.064	.949	[−.100, .092]
**SC → IWB**	Direct	.267	4.730	< .001	[.156, .377]
**BLM → IWB**	Direct	−.056	.935	.350	[−.172, .059]
**BLM → SC → IWB**	Indirect	−.085	3.839	< .001	[−.133, −.046]
**BLM → IWB**	Total	−.141	2.677	.007	[−.244, −.038]

Panel C.

Explained variance

Specification	Endogenous construct	R^2	Adjusted R^2
Meaningful-work model	MW	.105	.097
Meaningful-work model	IWB	.101	.096
Sense-of-community model	SC	.164	.157
Sense-of-community model	IWB	.085	.080

Note. The meaningful-work and sense-of-community specifications were estimated as two separate models. In each model, leader BLM, IOVC, and their interaction were entered simultaneously as predictors of the focal dimension. The focal dimension and leader BLM were entered as predictors of IWB. The indirect and total associations were estimated separately for each specification using 5,000 bootstrap resamples and two-tailed tests. The reported *t* statistics are absolute values.
